# The effects of simulation-based education on initial neonatal evaluation and care skills

**DOI:** 10.12669/pjms.35.4.350

**Published:** 2019

**Authors:** Ayse Karakoc, Meltem Demirgoz Bal, Fadime Bayri Bingol, Begum Aslan

**Affiliations:** 1 Ayse Karakoc, PhD. Assistant Professor, Department of Midwifery, Marmara University, Health Sciences Faculty, Istanbul, Turkey; 2 Meltem Demirgoz Bal, PhD. Associate Professor, Department of Midwifery, Marmara University, Health Sciences Faculty, Istanbul, Turkey; 3 Fadime Bayri Bingol, PhD. Assistant Professor, Department of Midwifery, Marmara University, Health Sciences Faculty, Istanbul, Turkey; 4 Begum Aslan, Research Assistant, Department of Midwifery, Marmara University, Health Sciences Faculty, Istanbul, Turkey

**Keywords:** Checklists, Delivery room management, Midwifery student, Newborn evaluation, Professional practice evaluation, Simulation training

## Abstract

**Objective:**

Neonatal evaluations performed at the very first minutes following postpartum are the most important steps in deciding for neonatal resuscitation. Therefore, the newborn initial care and evaluation notion and skills of midwives in the delivery hall are quite important. The study was planned to determine the effects of simulation education on newborn evaluation and care skills in midwifery students.

**Methods:**

This is a quasi-experimental study. The population of the study was composed of the 4th year students of Marmara University Faculty of Health Sciences (65 students in total), who selected the Intern Newborn course in the 2017-2018 Fall and Spring semesters.

**Results:**

The areas where the control group students did not apply at all or needed the help of the trainer were observed as delivery room preparation (86.2%), initial neonatal evaluation (96.6%) and registration/safety (69%). According to “the Guide for Newborn Evaluation at the Delivery Room,” the differences in the mean total scores and all sub-dimension scores were found to be significant in favor of the experiment group.

**Conclusion:**

Education programs that are carried out by computer-assisted simulation and in accordance with the teaching guide were effective on improvement of knowledge-skills on newborns’ first evaluations in the delivery room.

## INTRODUCTION

The very first minutes following the postpartum stage are a process involving various risks regarding the newborn in the short and long terms. For this reason, midwives, who are the most important members of the delivery team, should be prepared and trained against all risks, and this is important for the overall success of delivery room management.

Neonatal resuscitation training programme (NRP) is reported to help reducing intrapartum-related infant mortality by 30%, and therefore, it is recommended to support NRP and neonatal evaluation trainings.^1^ Neonatal evaluation performed at the very first minutes following postpartum are the most important steps in deciding for NRP. The time following the initial care and practice is described as the golden hours of the newborn.^2^ Therefore, the newborn’s initial care and evaluation notions and skills of midwives in the delivery hall are quite important.

Within the context of midwifery education, the initial care and follow-up of the newborn in the delivery room is a particularly important issue. To be able to care and follow up care of at least 100 healthy newborns is essential to be competent in the skills that need to be gained before graduation.^3^ In the case of risky newborns, it has been reported that simulation techniques used in skill development in line with neonatal resuscitation training in the delivery room increase experience and skill points.^1^,^4^-^8^

Simulation-based trainings provide the student with the opportunity to encounter and practice practical cases that are very close to reality in a safe environment. These are used in fields such as transfer of knowledge, skill development and evaluation, emergency training, orientation programs of new graduates, in-service training and evaluation of clinical competencies.^9^-^11^

Simulation-based training is recommended, especially as it increases patient safety for infants and children.^12^ There is an insufficient number of studies with simulation trainings regarding their effects for the initial evaluation/care of the healthy newborn in the delivery room. This study may contribute new information to the literature in this direction.

## METHODS

The study was planned as a quasi-experimental study to determine the effects of simulation-based education on the initial neonatal evaluation and care skills of midwifery students. The population of the study was composed of the 4th year students of Marmara University Faculty of Health Sciences (29 students in the Fall semester, 36 students in the Spring semester, 65 students in total), who selected the Intern Newborn course in 2017-2018 Fall and Spring semesters. Sample selection was not carried out, and all students who agreed to participate were included in the study.

While calculating the sample size, G. Power 3.0 analysis was used with a 0.5 effect size and 0.80 power, in a 95% confidence interval, and the number of active samples was found out to be at least 52. Considering data losses, the study was completed with a total of 65 participants (experiment: 36, control: 29).

### Ethics Approval and Permissions

The study was approved by the ethics committee of Marmara University Faculty of Health Sciences (dated 19.04.2018-9).

### Data Collection Tools

The data were collected by using a personal information form including the individual characteristics of the participants and the “Delivery Room Neonatal Initial Care/Evaluation Guide” (Appendix 1) as non-risk neonatal individuals were used.

**Table:** Supplemental Appendix-1:

Guide for First Care/Evaluation of the Newborn at the Delivery Room				
1. Not enough:	Did not perform the step, performed wrongly			
2. Needed improvement:	Did not perform the step in order, deficits and/or need help from the trainer			
3. Adequate/Expert	Performed the steps correctly and in order without stopping or help			

	*1*	*2*	*3*	*Min-max score*

*Preparedness for delivery*				
1.Taking antenatal anamnesis				9-27
2.Examining risk factors				
3.Set the delivery room at temperatures of >26C /control				
4.Opening radiant heater for the newborn 15 minutes before				
5.Preparing warm dry covers for the newborn				
6.Preparing resuscitation materials/control				
7.Preparing arm bands				
8.Preparing materials				
9.Determining the delivery team				
*First minute evaluation after birth*				
1.Newborn term				4-12
2.Respiratory effort				
3.Is tonus good				
4.HR (hearth rate)>100				
*Placenta/Umbilical cord*				
1.Wait with mother or low at least for 1 minute				2-6
2.Clamp the cord and cut				
*Applications/Interventions*				
1.Drying by starting with the head with warm cover and protecting vernix caseosa				5-15
2.Remove moisture quickly				
3.Swap in mouth and nose				
4.Supply skin-to-skin contact (place baby’s face directly on the mother, cover the baby’s back with a warm cover, cloth a hat for the baby) (at least 30 minutes-1 hour)				
5.If there is excessive secretion, place baby under radiant heater, give head position, aspire mouth at first, nose after (80-100 mmHg)				
*First examination (Short and fast evaluation)*				
1.Vital signs				9-27
2.General view				
3.Extremity movement (flexion, symmetry, movement)				
4.Birth trauma (caput succedaneum, cephalohematoma)				
5.Major congenital anomalies (omphalocele, neural tube defects, cleft lip, etc.)				
6.Length-weight-head circumference				
7.1 mg vitamin-K IM (vastus lateralis)				
8.Prophylactic eye drop				
9.Hepatitis B vaccine				
*Documents and security*				
1.Make the mother and the infant wear arm bands (name-surname, birth date, hour, birth weight)				4-12
2.Take footprint of the baby and fingerprint of the mother				
3.Record all implementation to file				
4.Supply breastfeeding within the first 30 minutes				

TOTAL				33-99

The personal information form included age, completed birth rate, newborn course grade and general weighted grade point average of the participants. The “Delivery Room Neonatal Initial Care/Evaluation Guide” for non-risk neonatal infants was prepared in accordance with the Turkish Neonatology Society’s Delivery Hall Management Guide, published by Oygur et al.^13^

Each step was evaluated with a 3-point Likert-type scale (1 = Not enough 2 = Developed, 3 = Sufficient). A minimum of 33 and a maximum of 99 points may be obtained from the guide. The higher score means more student’s knowledge and skills.

The simulator that was used in the study was supported by the Marmara University Scientific Research Projects Coordination Unit (SRPCU) within the scope of the infrastructure project titled “Establishment of Gynecology Simulation Unit for Improving Vocational Training Infrastructure of Midwifery Department” (Project No: SAG-E-090517-0244, 2018). The brand of the automated software simulation system was GAUMARD, while the model of the mock up was the Noelle, S554.100-Maternal and Neonatal Birthing Simulator.

### Process

The control group students consisted of those who registered for the intern neonatal course before the computer-aided simulation laboratory was established. In the control group, “Delivery Room Neonatal Initial Evaluation/Care” subject was instructed via power point presentations and verbally in line with the curriculum. After two weeks, the students were provided with the necessary explanations, and the students who agreed to participate in the study were evaluated in accordance with the Delivery Room Neonatal Initial Evaluation/Care Guide (Appendix.1). Preparedness for the delivery room, first-minute neonatal evaluation, umbilical cord clamping time, initial interventions, initial examination and recording-safety procedure steps were evaluated based on application of knowledge-skills.

In the experiment group, the “Delivery Room Neonatal Initial Evaluation/Care” subject was instructed via a Computer-Aided Simulation Model in accordance with the Newborn Evaluation Guide for the students registered for the Intern Neonatal Course. Simulation in the experiment group included first evaluation and care applications on newborns by demonstration to teach these skills to the students. Then, each student repeated the simulation 3 times under the supervision of the consultant. Two weeks later, a skill assessment was performed in line with the “Delivery Room Neonatal Initial Evaluation/Care” (Appendix.1). Preparedness for the delivery room, first-minute neonatal evaluation, umbilical cord clamping time, initial interventions, initial examination and recording-safety procedure steps were evaluated for application of knowledge-skills.

**Figure:**
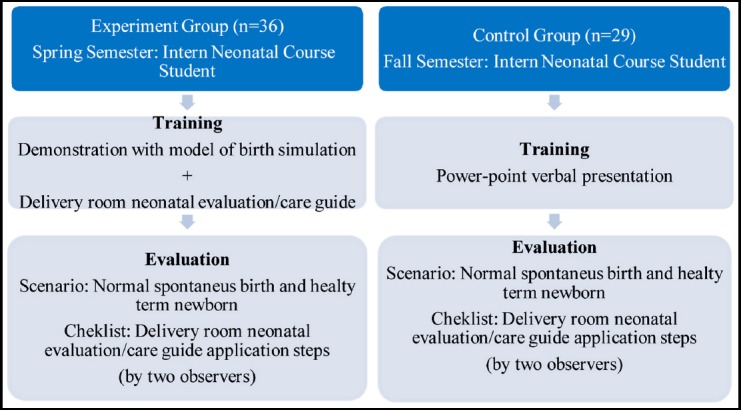
Study Schema

The students were evaluated in terms of their knowledge and skills using an objective structured evaluation method. A normal spontaneous birth (from third cycle) and healthy term neonatal scenario were designed for all participants in the computer-aided delivery simulation laboratory (Scenario: Mother: 35 years primipara, seamless pregnancy, vital signs normal, no intervention at birth, natural birth. Newborn: single baby, 3100 g, spontaneous breathing, pulse: 130, color: pink). Scoring was achieved in accordance with the “Delivery Room Neonatal Initial Evaluation/Care Guide.”

In the evaluation of the findings, besides the total score of the evaluation form, the total scores of both the sub-dimensions and the evaluation scores of each process step were compared between the groups. At the end of the study, the control group students were provided with the same level of training in accordance with the computer-aided simulation model and guideline to ensure an equal level of education ethically in between the participants.

### Statistical analysis

Both the experiment and the control groups’ knowledge and skill assessments for each student were conducted simultaneously and independently by two observers. The inter observer consistency was tested using Kendall’s Coefficient of Concordance and found to be 0.97. When inter observer consistency is between 0.80 and 1.00, it is considered to be high. Since this value was found to be quite high in the study, the scores of the second observer were used in the statistical analyses. It was found that the data were not normally distributed (Shapiro-Wilk, p <0.005). In the comparison of the experiment and control groups, non-parametric tests (Mann-Whitney U and Chi-Square) were applied based on the characteristics of the data.

### Limitation of the study

Within a year, the number of students taking the newborn intern course is limited. The simulation unit was established in 2018 in the spring period. Therefore, the sample size was limited to a total of 65 students (experiment: 36; control: 29).

## RESULTS

There was no significant difference between the experiment and control groups in terms of age (p=0.38), number of completed births (p=0.45), general weighted average (p=0.95) and newborn health academic success (p=0.93). It was found that the groups showed homogenous distribution (p>0.05).

The distribution of the scores obtained from the sub-dimensions of the Newborn Evaluation Guide is shown in Table-I. Additionally, the areas where the control group students did not apply at all or needed the help of the trainer were observed as delivery room preparation (86.2%), initial neonatal evaluation (96.6%) and registration/safety (69%) in accordance with the evaluations conducted in terms of the total scores of the sub-dimensions.

**Table I:** Distribution of mean scores of sub-dimensions in the newborn evaluation guide.

Preparedness for delivery room	Minimum	Maximum	X+SD
Experiment (n=36)	23	27	25.72±1.18
Control (n=29)	9	24	15.03 ± 3.95
*First-minute evaluation after delivery*			
Experiment (n=36)	8	12	11.53±0.89
Control (n=29)	4	10	4.41±1.35
*Umbilical cord/Placenta*			
Experiment (n=36)	4	6	5.69 ±0.39
Control (n=29)	2	6	5.10 ± 1.20
*Applications/interventions*			
Experiment (n=36)	12	15	14.56 ± 0.80
Control (n=29)	6	15	10.48 ± 2.40
*First examination*			
Experiment (n=36)	23	27	25.72 ± 1.03
Control (n=29)	11	27	19.07 ± 3.85
*Documentation/Security Measures*			
Experiment (n=36)	10	12	11.78 ± 0.48
Control (n=29)	4	11	7.17 ± 2.34
**TOTAL**			
Experiment (n=36)	91	99	95.17 ± 2.13
Control (n=29)	45	81	61.27 ± 8.12

The difference between the scores of the experiment and control groups regarding the dimensions of delivery room, initial neonatal evaluation, umbilical cord/placenta, operations/procedures, initial examination, registration/safety and total scores was found to be significantly higher in favor of the experiment group. Table-II

**Table II:** Comparison of scores in the guide for newborn evaluation at the delivery room.

Dimensions of Guide for Newborn Evaluation	Experiment (n=36) X ± SD	Control (n=29) X ± SD	Z*	p
Preparedness for delivery room	25.72±1.18	15.03 ± 3.95	-6.89	0.000
First minute after delivery	11.53±0.89	4.41±1.35	-7.31	0.000
Umbilical cord/Placenta	5.69 ±0.39	5.10 ± 1.20	-3.27	0.001
Applications/interventions	14.56 ± 0.80	10.48 ± 2.40	-6.64	0.000
First examination	25.72 ± 1.03	19.07 ± 3.85	-6.26	0.000
Documentation/Security Measures	11.78 ± 0.48	7.17 ± 2.34	-7.13	0.000

TOTAL	95.17 ± 2.13	61.27 ± 8.12	-6.90	0.000

Additionally, the results of each implementation step of the evaluation form were compared between the groups. The difference between the groups was statistically significant in all application steps (p<0.005), except for umbilical cord clamping (Chi-squared=1.360, p=0.505), vitamin-K vaccination (Chi-squared=2.56 p=0.109) and hepatitis vaccination (chi-square=2.56 p= 0.109).

## DISCUSSION

In the study, it was observed that the scores of the Neonatal Health course students considering both the experiment and the control group were quite high (mean: 85). This shows that their theoretical knowledge was quite adequate, but it also emphasizes a need for realistic simulation methods for clinical practice. Supporting the results of the study, it was reported that the students frequently demanded additional trainings related to their application skills in studies conducted with Health Sciences students.^14^,^15^

The study revealed that the participants in the control group got their lowest scores particularly from the delivery room preparation, initial neonatal evaluation and registration/security steps, while they especially either did not practice the process steps at all or needed the help of the instructor. Yoruk (2012), in his PhD dissertation on the skills of last year midwifery students, revealed in line with this study that the control group midwifery students either did not practice the process steps at all or needed the help of the instructor in terms of medical and obstetric history (100%), physical examination of the infant (38%) and recording/information processing (100%).^16^ In another study that evaluated the orientation training given to students who started their practice at the hospital, it was emphasized that students got high scores in communication subjects, whereas they received low scores in the patient safety/registration and infection control topics.^17^

As demonstrated in these studies which reported result in parallel to our study, there can be significant negligence regarding registration/security procedures. However, the most important factor that will protect midwives regarding both the sustainability of patient care processes and in possible malpractice cases is the recording of data.

Additionally, overlooking procedures such as obtaining antenatal history and preparation/control of materials within the scope of delivery room preparation may result in a loss of time in case of possible risks during delivery. Moreover, overlooking or a delay in the initial evaluation procedure of the newborn may have negative consequences for NRP decision-making processes.

The fact is that all the knowledge and skill scores of the experimental group students who were trained in accordance with the simulation and learning guide were significantly higher in line with the Neonatal Evaluation Guide, in parallel with the results of Kempster^6^, Carolan^7^, Williams^8^, Agraval^18^ and Durmaz.^19^

Additionally, no significant difference was encountered between the groups in evaluating the skills of clamping/cutting of the umbilical cord, vitamin-K and Hepatitis vaccination. This

situation suggests that the students attached more significance to the steps of the process that we can describe as physical intervention. Moreover, this may be due to the fact that these procedures are the most common practices that they have implemented during their clinical practice. It may be advisable to plan new studies involving applications that students practice most frequently in clinics.

In the light of the results of different studies and a systematic review, it may be reported that simulation-based trainings provide students with additional skills such as satisfaction, self-confidence, self-efficacy, critical thinking, team development, ability to communicate with team members and ability to take responsibility in stressful situations.^20^-^25^

## CONCLUSION

In order to develop the initial neonatal care/evaluation knowledge and skills of students in the delivery room, training in line with computer-aided simulation and learning guide is effective. Simulation-based training is a factor that increases the competency of a student.
